# Single-Molecule Long-Read Transcriptome Dataset of Halophyte *Halogeton glomeratus*

**DOI:** 10.3389/fgene.2017.00197

**Published:** 2017-12-01

**Authors:** Juncheng Wang, Lirong Yao, Baochun Li, Yaxiong Meng, Xiaole Ma, Huajun Wang

**Affiliations:** ^1^Gansu Provincial Key Lab of Aridland Crop Science, Gansu Key Lab of Crop Improvement and Germplasm Enhancement, Gansu Agricultural University, Lanzhou, China; ^2^Department of Crop Genetics and Breeding, College of Agronomy, Gansu Agricultural University, Lanzhou, China; ^3^Department of Botany, College of Life Sciences and Technology, Gansu Agricultural University, Lanzhou, China

**Keywords:** Halophyte, *H. glomeratus*, Iso-Seq, transcriptome, salt stress

## Introduction

Soil salinization has become a major challenge for sustainable development of global agriculture. As a result, cultivation of salt-tolerant crop varieties has become a focus of plant breeding. However, development of effective breeding strategies would be significantly enhanced by improving our understanding of salt tolerance mechanisms in plants and identifying genes required for adaptation (Flowers and Colmer, 2008; Shabala, 2013; Ismail and Horie, 2017).

Na^+^ is not essential for plant survival; in fact, there are many halophytes whose growth is improved upon the addition of a certain degree of NaCl (Bose et al., 2014). As a result of this unique survival strategy, halophytes have been used to elucidate a series of mechanisms that use to cope with salt stress. In particular, succulent halophytes, a representative type of anatomical adaptation in plants by increasing cell size due to the expansion of vacuole volume under salinity, have been suggested to take advantage of tissue- and organelle-specific ion compartmentalization to maintain high K^+^/Na^+^ ratios in response to high salinity (Munns and Tester, 2008; Shabala and Mackay, 2011). Furthering our molecular understanding of the ion transport systems and compartmentalization mechanisms used by halophytes is crucial for mapping the complex salt tolerance network in plants in order to optimize breeding of salt-tolerant crops.

*Halogeton glomeratus* (*H. glomeratus*), an annual, succulent halophyte, is a typical salt-tolerant halophyte with highly succulent leaves that has been used to characterize the physiological, cellular, and molecular mechanisms involved in the response to salt stress (Wang et al., 2015a,b, 2016).

The majority of next-generation transcriptome sequencing (NGS) technologies, or RNA sequencing (RNA-Seq) are not capable of assembling full-length transcripts due to the short lengths of sequencing-reads, which are likely to produce partial reference transcriptomes (Martin and Wang, 2011). For the RNA-Seq of non-model organisms, one of the principal strategies is *de novo* assembly. In this way, we could retrieve plenty of transcripts by piecing together short, low-quality sequencing-reads in a more economical approach, and provide a new opportunity to cognize the transcriptome characteristics of non-model organisms, but the disadvantages of the *de novo* assembly are obvious, such as a greater sequencing depth, highly sensitive to sequencing errors, presence of chimeric molecules in the sequencing dataset, and difficult to distinguish highly similar transcripts (Martin et al., 2010; Martin and Wang, 2011). In a word, it is still challenging to construct of a comprehensive transcriptome from short sequencing-reads. However, recent development of Pacific Biosciences (PacBio) long-read technologies for transcriptome sequencing (Iso-Seq) can overcome those limitations to capture the full catalog of transcripts and their variants (Sharon et al., 2013; Minoche et al., 2015). Thus, for the first time, we applied the Iso-Seq protocol to analyze the *H. glomeratus* transcriptome. These findings serve to improve *H. glomeratus* transcriptome annotation and provide abundant gene resources related to improve salt tolerance in plants.

## Value of the data

1. *Halogeton glomeratus* is not only highly valuable for the characterization of salt tolerance mechanisms in halophytes, but may also serve as a source of stress tolerance genes for plant breeding.
2. So far, there is lack of well-characterized transcriptomic profiling of *H. glomeratus*. Here, we provided the first comprehensive transcriptome analysis using single-molecule sequencing.
3. These data will help elucidate salt tolerance mechanisms of *H. glomeratus* and provide salt-tolerant gene resources for developing stress-tolerant plants.

## Data

### Transcriptome plant materials and NaCl treatments

*Halogeton glomeratus* seedlings were grown in plastic pots in a growth chamber and were irrigated daily with half-strength Hoagland's nutrient solution as previously described by Wang et al. (2015a). One month after germination (August 10, 2016), two independent experiments were conducted to assess different durations of salt stress. For the first experiment, seedlings were transferred to half-strength Hoagland's nutrient solution supplemented with 0, 100, 200, or 400 mM NaCl for a period of 3 days. The leaf samples were collected for each condition. For the second experiment, the seedlings were transferred to half-strength Hoagland's nutrient solution supplemented with 200 mM NaCl for 0, 2, 6, 24, or 72 h. The root samples were collected for each condition and were rinsed with distilled water. All samples were frozen in liquid N_2_ immediately.

### Generation of a barcoding library and single-molecule sequencing

After grinding the tissue samples, total RNA was extracted from each sample using the RNeasy Plant Mini kit (Qiagen; Hilden, Germany) and quantified using an Agilent 2100 bioanalyzer. The total RNA from the 9 samples were pooled for sequencing of an *H. glomeratus* reference transcriptome using single-molecule long-read technology (Iso-Seq) via the PacBio RS II sequencing platform.

In brief, total RNA (10 ng) was reverse transcribed using the SMARTer PCR cDNA Synthesis Kit (Clontech; Mountain View, CA, USA). After polymerase chain reaction (PCR) optimization, large-scale PCR reactions were performed to synthesize second-strand cDNA, which was subjected to BluePippin™ size selection using the following bins: 1–2, 2–3, 3–6, and 5–10 kb. After size selection, another round of amplification was performed using 12 PCR cycles. The amplified and size-selected cDNA products were used to generate SMRTbell template libraries for sequencing based on the Iso-Seq protocol. A total of 12 SMRT cells (Table 1) were sequenced on a PacBio RS II platform (Completed on January 2, 2017). Raw sequence reads (FASTQ format) are available through the NCBI database (https://www.ncbi.nlm.nih.gov/) with the following accession information (Data was released on September 22, 2017, size is 1.6 Gb):

BioProject ID: PRJNA359784.
BioProject ID: SAMN06298282.

**Table 1:** Proportion of SMRT reads containing cDNA primers and poly(A) tail and their classification.

**Database**	**Cell number**	**All reads**	**Five prime reads (%)**	**Three prime reads (%)**	**Poly-A reads (%)**	**Full-length non-chimeric reads (%)**	**Full-length non-chimeric read length (bp)**
1–2 kb	4	152,748	68.01	69.89	69.12	57.96	1,657
2–3 kb	4	176,650	60.10	62.56	62.02	50.05	2,848
3–6 kb	2	52,148	72.13	74.09	73.44	58.28	3,835
5–10 kb	2	51,874	53.04	61.68	59.97	35.33	6,221

### Bioinformatics analysis of isoform sequencing

Raw SMRT sequencing reads were classified as full-length (FL) non-chimeric, FL chimeric, non-FL, or short reads based on detection of 5′- and 3′-primer and polyA-tail sequences using SMRT Analysis v2.3.0 (http://smrt-analysis.readthedocs.io/en/latest/SMRT-Analysis-Software-Installation-v2.3.0/). Only reads with both 5′- and 3′-primers and polyA-tail sequences were considered to be FL. FL non-chimeric reads were clustered into consensus sequence (CS) reads using the ICE (Interative Clustering and Error Correction) algorithm and further polished using Quiver. Finally, high quality CS reads from each library were merged, and redundancy was removed to identify unique isoforms present in the *H. glomeratus* transcriptome using the Cd-hit program (Li and Godzik, 2006) for further analysis. All of the isoform sequences are publicly available on Figshare at https://figshare.com/articles/Single-molecule_long-read_transcriptome_dataset_of_halophyte_Halogeton_glomeratus/5345464.

**Figure 1:**
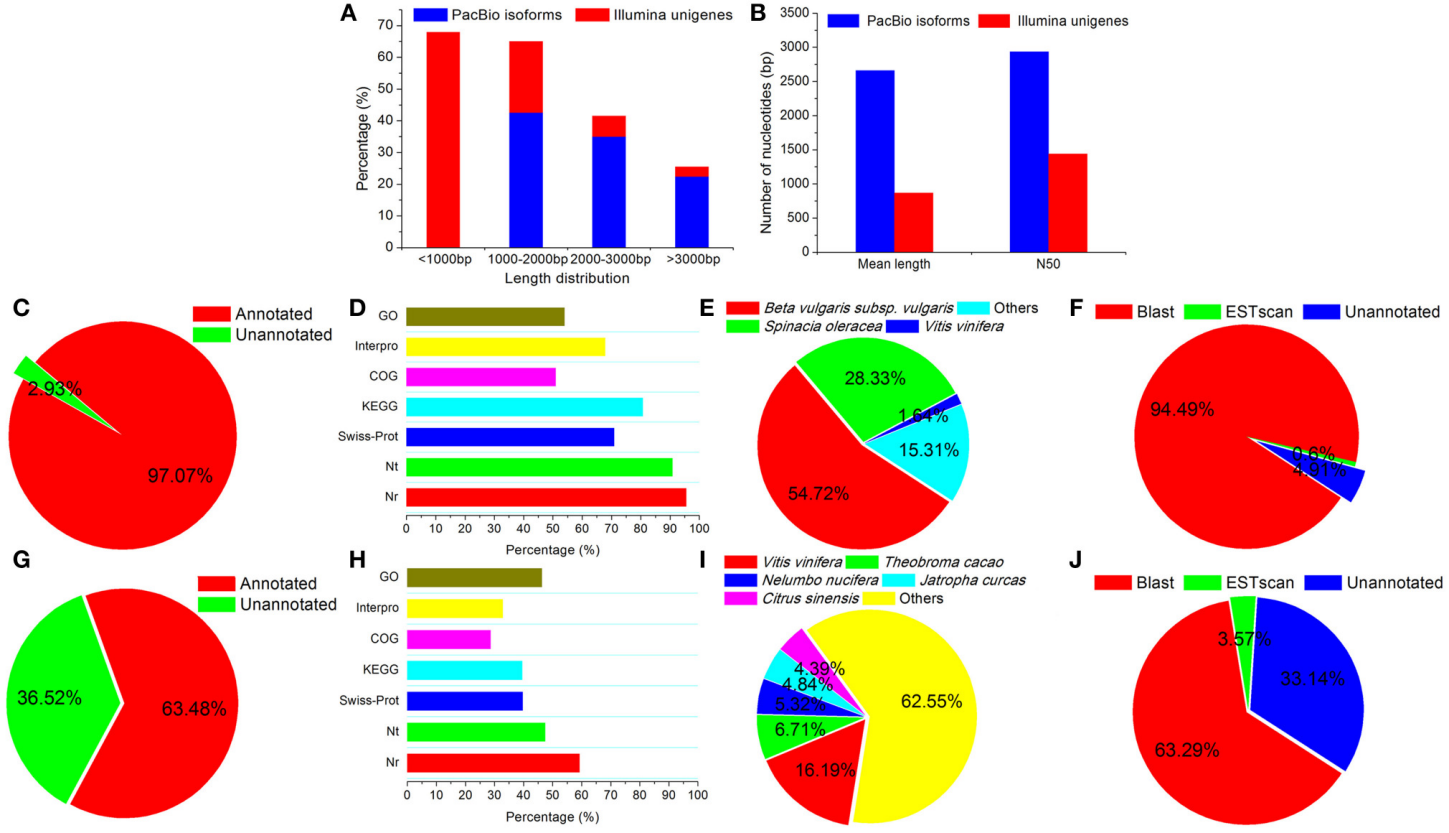
Comparison of transcriptome results from Illumina and PacBio sequencing platforms. Transcript length distribution **(A)**, mean length **(B)**, percentages of functional annotation **(C,G)**, percentages of NR, NT, GO, COG, KEGG, Swissprot, and Interpro annotation **(D,H)**, species distribution for NR annotation **(E,I)**, and CDS predictions **(F,J)** were compared for the PacBio **(A–F)** and Illumina **(A,B,G–J)** sequencing platforms.

A total of 433,420 reads of insert (ROIs) were generated. Full-length (FL) non-chimeric transcripts made up 57.96% of the 1–2 kb transcripts, 50.05% of the 2–3 kb transcripts, 58.28% of the 3–6 kb transcripts, and 35.33% of the 5–10 kb transcripts, and the libraries were characterized by average lengths of 1,657, 2,848, 3,835, and 6,221 bp, respectively (Table 1). The FL non-chimeric transcripts were processed using Quiver to yield 64,910 high quality consensus isoforms. After additional merging and discarding of redundancies, we obtained 54,835 consensus isoforms with a mean length of 2,663 nt and a N50 length of 2,937 nt (Figures 1A,B).

The isoform sequences were used as queries for sequence homology searches (*E* < 0.0001) performed with Blast, Blast2GO (Conesa et al., 2005), and InterProScan5 (Quevillon et al., 2005) to identify functional annotation terms from the non-redundant protein (NR), non-redundant nucleotide (NT), Gene Ontology (GO), Clusters of Orthologous Groups (COG), Kyoto Encyclopaedia of Genes and Genomes (KEGG), SwissProt, and Interpro databases. For isoform coding DNA sequence (CDS) predictions, we selected the segment of the isoform that best mapped to the functional databases as its CDS. For isoforms that were not annotated in the databases, we used ESTScan (Iseli et al., 1999) to predict CDSs. The predict CDSs of isoforms are publicly available on Figshare at: https://figshare.com/articles/Single-molecule_long-read_transcriptome_dataset_of_halophyte_Halogeton_glomeratus/5345563.

A total of 53,230 (97.07%) isoforms were matched to known genes, and 52,141 (95.07%) isoforms were identified to have intact coding DNA sequences (CDS) (Figures 1C–F). At the same time, our previous Illumina RNA-Seq data of *H. glomeratus* (Wang et al., 2015b) was re-annotated by the update versions of the database used in the present Iso-Seq transcriptome sequencing, including NR, NT, GO, COG, KEGG, SwissProt, and Interpro databases. In comparison to the results from Illumina RNA-Seq analysis of *H. glomeratus*, the length distribution, functional annotation, and coding sequence quantity of the Iso-Seq transcripts were significantly improved (Figure 1). In particular, with respect to the species distribution of annotation from the NR database, we found that 98.31% of the annotated isoforms showed the highest similarity to sequences from the three most prevalent species: *beta vulgaris* subsp. *vulgaris* (54.72%), *spinacia oleracea* (28.33%), and *vitis vinifera* (15.31%) (Figure 1E). Like *H. glomeratus, beta vulgaris* subsp. *vulgaris* and *spinacia oleracea* belongs to *Chenopodiaceae*. In contrast, for unigenes identified using Illumina RNA-Seq, the top species identified was *vitis vinifera* (16.19%), and none of the top five species belong to *Chenopodiaceae* (Figure 1I). Besides, to better understand the advantages of the PacBio Iso-Seq reference transcriptome, we mapped the Illumina *de novo* assembled transcripts (50,267) to the Iso-Seq consensus transcripts (54,835). Among Iso-Seq consensus transcripts, a total of 21,008 exhibited homology to Illumina *de novo* assembled 36,548 transcripts (*E* < 0.0001), of which 8,236 (39.20%) exhibited homology with at least two Illumina *de novo* assembled transcripts, including 4,668 (22.22%), 1,860 (8.85%) and 1,708 (20.74%) exhibited homology with two, three, and the more Illumina *de novo* assembled transcripts, respectively (Supplementary Figure 1). These results are successfully consistent with comprehensive assembly and annotation of a reference *H. glomeratus* transcripts using Iso-Seq technology that is substantially more contigous and contains a higher proportion of intact CDS.

## Author contributions

HW designed the experiments. JW, LY, BL, YM, and XM collected the samples and performed the experiments. JW, LY, and BL analyzed RNA-Seq data. JW and LY wrote the manuscript. All authors read and approved the final manuscript.

### Conflict of interest statement

The authors declare that the research was conducted in the absence of any commercial or financial relationships that could be construed as a potential conflict of interest.

## Supplementary material

Supplementary Figure 1 Statistics of the correspondence between the Illumina unigenes and PacBio reference transcripts of *H. glomeratus* transcriptome.
