# Supplementary figures and images for: Single-Molecule Long-Read Transcriptome Dataset of Halophyte Halogeton glomeratus
Source: Front Genet. 2017 Dec 1;8:197. doi: 10.3389/fgene.2017.00197 (PMC5716979; doi:10.3389/fgene.2017.00197)

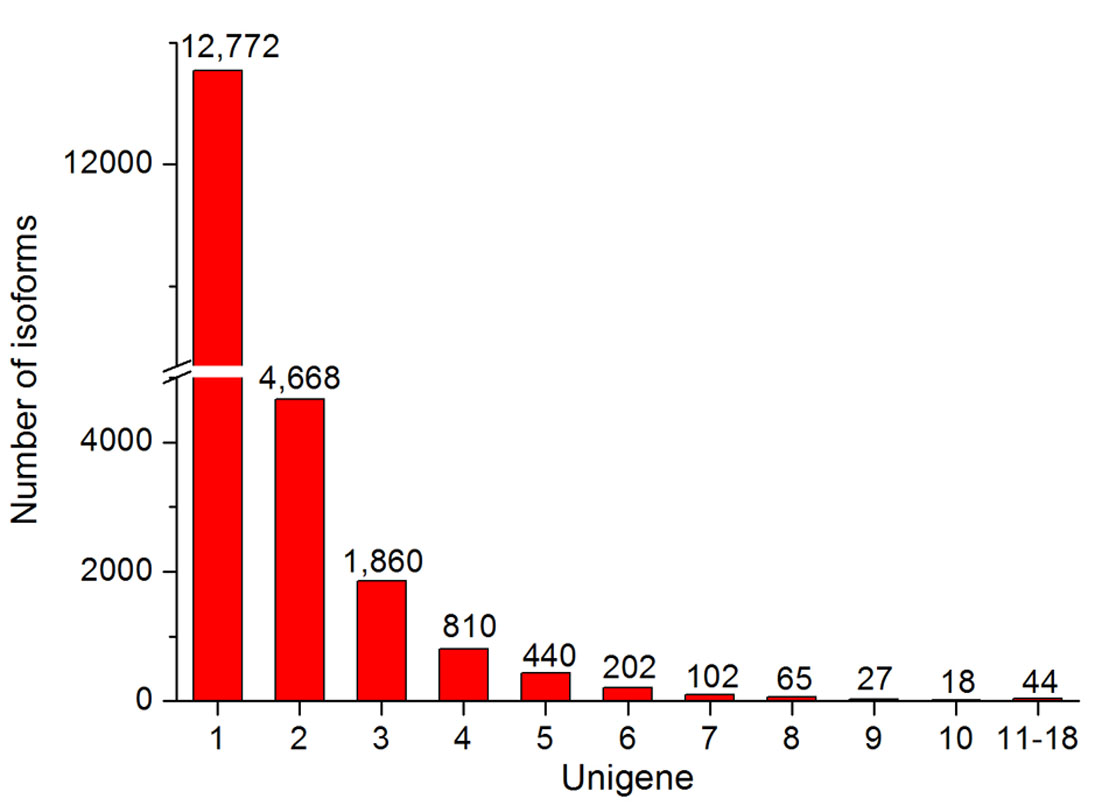

Supplement: Supplementary Figure 1 — Statistics of the correspondence between the Illumina unigenes and PacBio reference transcripts of H. glomeratus transcriptome. [file Image1.JPEG]
